# Decoding the formation of barred olivine chondrules: Realization of numerical replication

**DOI:** 10.1126/sciadv.adw1187

**Published:** 2025-05-23

**Authors:** Hitoshi Miura, Tomoyo Morita, Tomoki Nakamura, Kana Watanabe, Akira Tsuchiyama, Yuki Kimura, Chihiro Koyama

**Affiliations:** ^1^ Graduate School of Science, Nagoya City University, Yamanohata 1, Mizuho-cho, Mizuho-ku, Nagoya 467-8501, Japan.; ^2^ Department of Earth Science, Tohoku University, 6-3, Aramaki-Aza-Aoba, Aoba-ku, Sendai 980-8578, Japan.; ^3^ State Key Laboratory of Deep Earth Processes and Resources, Guangzhou Institute of Geochemistry, Chinese Academy of Sciences, Guangzhou 510640, China.; ^4^ Research Organization of Science and Technology, Ritsumeikan University, Shiga 525-8577, Japan.; ^5^ Institute of Low Temperature Science, Hokkaido University, Kita-19, Nishi-8, Kita-ku, Sapporo 060-0819, Japan.; ^6^ Human Spaceflight Technology Directorate, Japan Aerospace Exploration Agency, 2-1-1, Sengen, Tsukuba-shi, Ibaraki 305-8505, Japan.

## Abstract

Millimeter-sized silicate spherules embedded in primitive meteorites, namely, “chondrules,” are the primary solid component of the early solar nebula. They exhibit distinctive solidification textures, formed through rapid cooling from a molten state. The formation conditions of these textures have primarily been inferred on the basis of dynamic crystallization experiments; however, the theoretical verification of the solidification process has been largely neglected. Here, we conducted numerical simulations of the solidification of chondrule melt and successfully reproduced a crystal growth pattern resembling a typical barred olivine chondrule texture. This pattern emerged under conditions of rapid cooling, exceeding 10^4^ kelvins hour^−1^, which is substantially larger than those inferred experimentally. These results suggest that theories of chondrule formation in the nebula, which have been developed based on experimental results, should be reexamined.

## Teaser

The solidification process of chondrules, the building blocks of planets, was mimicked by sophisticated numerical simulations.

## INTRODUCTION

Chondrules, comprising 20 to 80 vol % of the volume of primitive meteorites known as chondrites (*1*), are a major solid component of the early solar nebula. Recent discoveries of chondrule-like objects in samples from the asteroids Ryugu and Bennu (*2*–*4*), as well as the discovery of similar objects in comets and other outer solar system bodies (*5*, *6*), underscore their widespread distribution. In planetary formation theory, millimeter- to centimeter-sized rocky debris, or “pebbles,” including chondrules, are believed to play a crucial role in the growth of planetesimals into protoplanets and giant gas planets (*7*, *8*). Understanding the formation mechanism of chondrules is key to elucidating the initial stages of planetary formation, where submicron-sized interstellar dust evolves into kilometer-scale planets. Despite their importance, the astronomical phenomena responsible for chondrule formation remain unresolved. Elucidating this mechanism is a vital research goal in planetary science.

Chondrules exhibit a variety of solidification textures, formed through rapid cooling from a molten state. The majority display porphyritic textures, consisting of multiple phenocrysts embedded in a glassy or microcrystalline mesostasis, while a few chondrules exhibit non-porphyritic textures, such as barred olivine (BO) and radial pyroxene (*9*). These textures have been reproduced in numerous ground-based dynamic crystallization experiments, and the conditions necessary for their formation have been investigated (*10*, *11*). These formation conditions have been established as “observational constraints.” Previous theoretical studies on chondrules primarily focused on identifying heating mechanisms that align with these observational constraints (*12*–*14*). However, with a few exceptions limited to simplified single-component systems (*15*, *16*), the theoretical exploration of solidification texture formation is scarce. Considering that determining formation conditions is fundamental to chondrule research, these conditions must be validated through both experimental and theoretical approaches. In this study, we conducted the numerical simulation of the solidification of chondrule melt, using a numerical calculation method based on a phase-field model (*17*, *18*) that considers a realistic multicomponent system. We aimed to reproduce a distinctive solidification texture exhibited by chondrules, namely, the BO texture.

## RESULTS AND DISCUSSION

### Selective evaporation from the chondrule melt surface

**Fig. 1.**
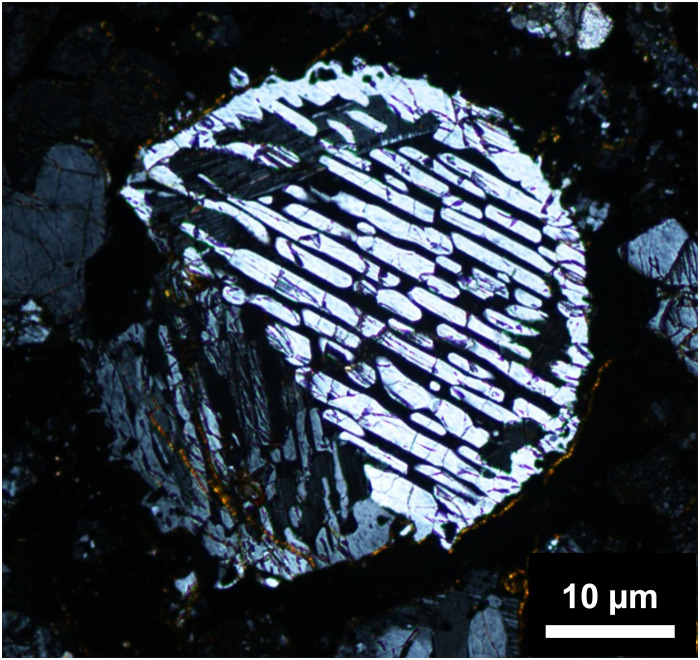
Typical texture of a natural BO chondrule from Asuka 97346 (CO3.3): microscopic image under transmitted polarized light. The texture comprises parallel bars of the olivine set and is surrounded by the olivine shell (rim). The bar and rim crystals are optically continuous, indicating a single crystal. The sample was provided by the National Institute of Polar Research.

**Fig. 2.**
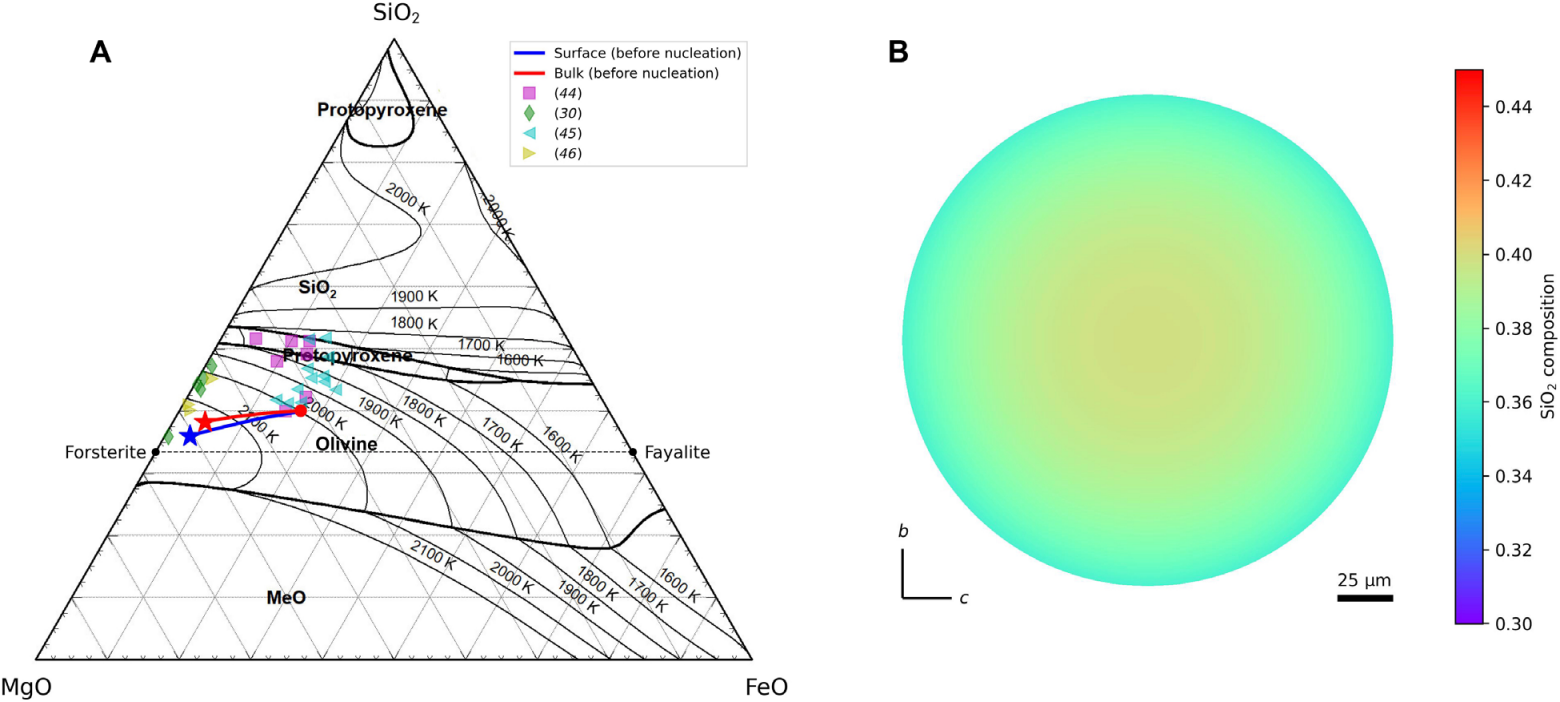
Composition changes in chondrule melts owing to evaporation. (**A**) MgO-FeO-SiO_2_ ternary diagram expressed in mole fraction. The red circle represents the initial composition of the chondrule melt before evaporation. The red line shows how the bulk composition changes as evaporation progresses. The blue line indicates how the composition of the melt surface changes. The star marks the composition of the melt at the onset of crystallization. The temperature labels along the lines represent the liquidus temperature of the melt. The dashed line represents the compositional range of Mg-Fe olivine solid solution. The phase diagram was created using FactSage (*43*). The symbols represent the bulk compositions of natural BO chondrules (*30*, *44*–*46*). (**B**) Distribution of SiO_2_ within the melt at the onset of crystallization.

These BO textures, characterized by a double structure of parallel bar–shaped olivines surrounded by a rim-shaped olivine in two-dimensional cross-sections (see Fig. 1), are believed to form through rapid solidification from a fully molten state (*11*). The high peak temperatures during melting likely lead to mass loss owing to evaporation, with the extent of loss varying depending on the chemical components in the melt. For FeO-MgO-SiO_2_-CaO-Al_2_O_3_ melts, FeO is the most susceptible to evaporation, followed by SiO_2_ (*19*). Selective evaporation from the melt surface induces compositional gradients within the melt (fig. S1). In this study, we consider a spherical melt droplet with a uniform equilibrium composition at 2000 K before evaporation. As illustrated in Fig. 2, the melt cooling at a rate of 10 K s^−1^ for 1 s experiences a substantial loss of FeO through evaporation, leading to an overall FeO-poor composition. Furthermore, the melt surface exhibits a marked depletion of SiO_2_ compared with the bulk composition, reflecting the slower diffusion of SiO_2_ relative to FeO (*20*). Owing to selective evaporation, the liquidus temperature at the melt surface increased to ~2150 K. Additionally, the SiO_2_ composition at the melt surface closely resembles that of olivine (fig. S2). This similarity in SiO_2_ composition between the melt and olivine has crucial implications for BO formation, as discussed below.

### Numerical simulations of the solidification of chondrule melt

**Fig. 3.**
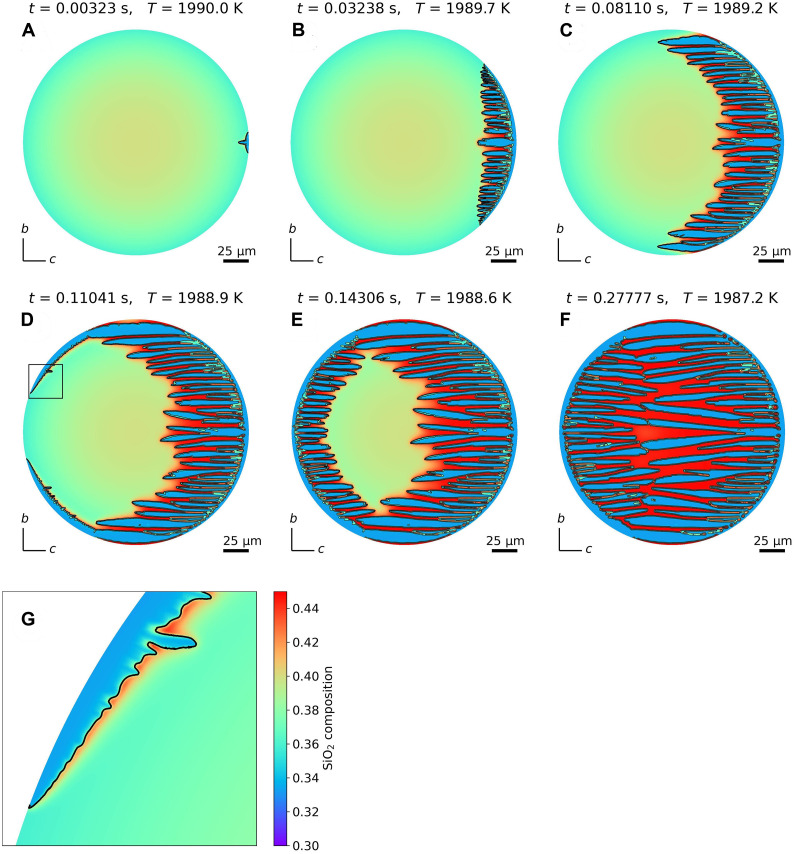
Numerical simulation of olivine crystal growth in a chondrule melt. A two-dimensional simulation of olivine crystal growth, perpendicular to the *a* axis. The color scale represents the SiO_2_ mole fraction [see (G)]. (**A**) Crystal growth immediately after the introduction of a seed crystal on the right side of the melt. (**B**) Rim and bar structures form along the right-hand side of the melt surface. (**C**) Many bars elongate along the *c* axis while maintaining a roughly parallel orientation to each other. (**D**) Some bars reach the left-hand side of the melt surface, and a rim structure forms along the surface. (**E**) Rim and bar structures form along the left-hand side of the melt surface. (**F**) The bars that have grown from both the left and right sides collide near the melt center, and the growth of olivine crystals is almost complete. (**G**) Enlarged image of the square area in (D). Notably, the SiO_2_ that could not be incorporated into the olivine is accumulated near the solid-liquid interface.

Figure 3 shows the results of numerical simulations of the subsequent crystal growth process, assuming the formation or attachment of an olivine crystal nucleus at a specific point on the melt surface where SiO_2_ was particularly depleted (evaporation layer). Supercooling on the melt surface is up to ~160 K with an elevated liquidus temperature. Initially, the crystal nucleus rapidly grows along the melt surface, corresponding to the formation of an olivine rim observed in many BO textures. Subsequently, numerous protuberances emerge inside the rim and elongate along the *c* axis while maintaining a roughly parallel orientation. This aligns with the parallel olivine bars observed in BO textures. The elongation along the *c* axis is attributed to the strong growth anisotropy of the olivine crystal, which inhibits growth along the *b* axis (*18*). Some bars eventually reach the left side of the melt surface, initiating the formation of a previously unidentified rim and bars. This calculation result is consistent with observations (*21*), suggesting that part of the rim forms using the bars as a substrate. Last, bars growing from both the left and right sides collide near the melt center, nearing the completion of olivine growth. The formation of the rim takes ~0.1 s to cover the entire circumference, whereas bar growth almost completes in ~0.3 s. The crystal growth rate can reach several hundred micrometers per second, and such rapid growth has been experimentally verified for olivine growth in a highly supercooled state using levitation methods (*22*–*25*). A magnified image of the growth tip of the rim suggests that interfacial instability (*26*) owing to local concentration gradients near the solid-liquid interface triggers bar formation. The rim and bars formed during this process originate from the same single crystal, ensuring consistency in crystallographic orientation. This aligns with the optical observation characteristics of BO textures (*21*, *27*). The remaining red area will eventually solidify into mesostasis comprising glass and a small amount of microcrystals.

The substantial suppression of growth along the *b* axis of the bar is assumed to be attributed to the extremely low density of kinks, which are sites for incorporating growth units, on the (010) face of olivine owing to its atomistic-scale flat structure (*18*). Nevertheless, the rim rapidly grew along the *b* axis at the melt surface, namely, a process considered to be driven by compositional changes in the surface evaporation layer. As shown in Fig. 2, the liquidus temperature increases owing to compositional changes in the surface evaporation layer, leading to an enhanced degree of compositional supercooling. This increased supercooling can induce atomic-scale structural changes in the solid-liquid interface, potentially increasing the kink density (*28*). This relaxation of the growth suppression along the *b* axis is incorporated into the numerical calculations by assuming reduced anisotropic properties of olivine near the melt surface (Materials and Methods). Notably, without considering this reduction in anisotropy, the calculations fail to establish the rim structure (fig. S3). This suggests that increased local compositional supercooling at the melt surface is a crucial factor for both the formation of highly anisotropic bars and development of a global-scale rim.

In addition to this factor, the similarity in SiO_2_ composition between the melt and olivine (compositional compatibility) is considered to have further promoted rim formation. Although olivine has a stoichiometric composition of 33 mol % of SiO_2_, the initial melt in our assumption is SiO_2_ rich compared with olivine. Consequently, the excess SiO_2_ not incorporated into olivine accumulates on the solid-liquid interface. Considering the relatively slow diffusion of SiO_2_, this accumulation substantially inhibits crystal growth. If the SiO_2_ composition of the melt was to perfectly match that of olivine, then this accumulation of SiO_2_ would not occur. As depicted in Fig. 2A, selective evaporation reduces the SiO_2_ concentration on the melt surface that closely approximates that of olivine. Although not a perfect match, this suppressed SiO_2_ accumulation mitigates the inhibitory effect on crystal growth. Notably, in this study, a numerical calculation method that is capable of accurately representing stoichiometric compounds, such as olivine, was used (*17*). Therefore, it reproduces the influence of compositional compatibility on crystal growth processes. The SiO_2_ compositional compatibility between the melt and olivine within the evaporation layer is considered to further promote crystal growth along the melt surface, contributing to the formation of the global-scale rim.

There are two groups of bulk compositions of BO: MgO rich and FeO rich (Fig. 2A). The fact that similar textures can form from different bulk compositions suggests the existence of a BO formation mechanism that is independent of the FeO content in the starting material. The rim formation mechanism proposed in this study, based on selective SiO_2_ evaporation at the melt surface, is essentially independent of the FeO content and can therefore be applied to both groups.

The role of selective evaporation on the melt surface in rim formation has been highlighted in the past. BO chondrule textures with the global-scale rim were replicated in crystallization experiments under vacuum conditions; rim formation was attributed to FeO evaporation, wherein the latent heat of evaporation decreases the temperature near the melt surface (local enhanced supercooling) (*29*). In contrast, our model assumes a uniform melt temperature, neglecting the latent heat cooling. We propose an alternative mechanism for global-scale rim formation on the basis of two key factors arising from selective SiO_2_ evaporation on the melt surface: an increase in the local liquidus temperature, and SiO_2_ compositional compatibility between the liquid phase and olivine.

### Chemical and isotopic composition

**Fig. 4.**
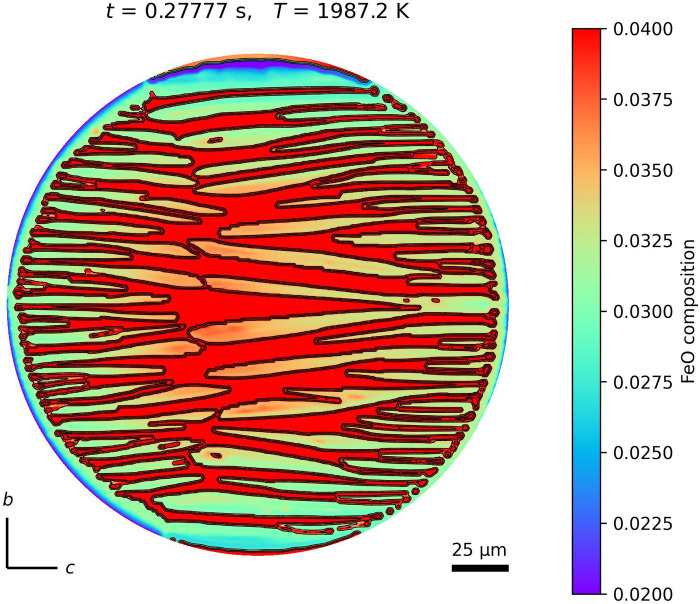
FeO zoning in olivine at the final stages of olivine growth. The color scale represents the FeO mole fraction. Red areas indicate the residual melt.

The olivine within BO chondrules exhibits heterogeneity in FeO composition, particularly pronounced in FeO-poor chondrules. The SD of FeO composition in some BO chondrules can reach several wt % (*27*). To investigate the cause of this heterogeneity in FeO composition, the calculated results of FeO distribution within olivine at the near-completion stage of olivine growth are shown in Fig. 4. The overall trend reveals a FeO-poor composition near the sphere surface and FeO-rich composition closer to the center. The FeO-poor composition near the surface is likely attributed to FeO depletion owing to evaporation. The FeO-rich composition in the center is probably attributed to the weaker influence of evaporation compared with that at the surface, as well as the gradual accumulation of FeO that cannot be incorporated into olivine within the residual melt. Notably, the FeO-rich region coincides with the meeting point of bars growing from both the left and right sides at the end of olivine growth. According to the calculation results, a composition difference of ~1 mol % occurs within the olivine single crystal. To verify our hypothesis, we must measure whether a systematic spatial change exists in the olivine composition distribution.

Our theoretical scenario is generally consistent with the chemical and isotopic compositions of BO chondrules reported to date. The olivine within BO chondrules exhibits a bimodal distribution of FeO composition (*27*). This phenomenon may also be explained by FeO evaporation. FeO-poor precursors require higher temperatures for complete melting owing to their high liquidus temperature, leading to increased FeO evaporation and a more FeO-poor composition. Conversely, FeO-rich precursors have lower melting temperatures, suppressing evaporation and maintaining an FeO-rich composition. This explanation qualitatively accounts for the occurrence of intermediate gaps in the FeO composition. In addition, selective evaporation would cause the melt, before nucleation, to become enriched in refractory components such as Al_2_O_3_ and CaO (*19*), while simultaneously depleting volatile elements such as Na. This trend aligns with the observation that glass inclusions in BO chondrules are rich in Al_2_O_3_ and CaO and that Ca and Na exhibit a negative correlation (*30*). Regarding isotopic composition, mass-dependent isotopic fractionation is thought to occur as a result of evaporation from the melts, with lighter isotopes, such as ^28^Si, being preferentially lost. This is consistent with measured data showing that BO chondrules tend to have high δ^30^Si (*31*) and with the hypothesis that δ^30^Si heterogeneity in chondrules arises from nonequilibrium evaporation-condensation processes during chondrule formation (*32*). On the other hand, the fact that isotopic composition changes in major elements such as Si, Mg, and Fe are limited to ~1.5 per mil (*31*) imposes a constraint on selective evaporation. In our theoretical model, the compositional change of the melts required for rim formation is confined to the vicinity of the melt surface and does not necessitate large-scale evaporation of the bulk. Therefore, mass-dependent isotope fractionation in the bulk may have been suppressed. A quantitative examination of chemical and isotopic composition changes during the crystallization of the melt is needed in future studies.

### Theoretical constraints

On the basis of our hypothesis that compositional gradients at the melt surface resulting from evaporation are crucial for BO chondrule formation, we can impose theoretical constraints on the cooling rates experienced by chondrules. First, the evaporation of SiO_2_ must be fast enough to establish a local compositional gradient at the melt surface. However, to prevent the complete depletion of SiO_2_, the duration of evaporation must be limited to $t<0.1a^{2}/D$ (Materials and Methods), where a is the chondrule radius and D is the liquid-phase diffusion coefficient. In particular, cooling from the peak temperature to the crystal nucleation temperature must be sufficiently rapid. Defining nucleation supercooling $\Delta T_{n}$ as the temperature difference between the peak and crystal nucleation temperatures, the required cooling rate can be estimated as $R_{c}=\Delta T_{n}/t≳10D\Delta T_{n}/a^{2}$. In dynamical crystallization experiments using melt levitation techniques, the melts exhibited a substantial resistance to crystallization, remaining in the liquid phase even after being cooled by several hundred kelvins below the liquidus temperature following complete melting (*22*–*25*). These observations suggest a substantial supercooling barrier, likely necessitating $\Delta T_{n}≳100\;K$. Substituting *D* = 10^−9^ m^2^ s^−1^ (*18*, *33*) and *a* = 1 mm, we obtain the condition $R_{c}≳3600\;K\;{\text{hour}}^{-1}$. This exceeds the previously suggested cooling rate range of 500 to 3000 K hour^−1^ for BO chondrules (*11*). Furthermore, as the required cooling rate is inversely proportional to the square of the chondrule radius, smaller BO chondrules necessitate even more rapid cooling. This represents a novel theoretical constraint derived from numerical simulations of chondrule-melt solidification.

The theoretical constraints proposed in this study apply to the temperature range from the fully molten state to the introduction of the first crystal nucleus. It is important to note that no strict constraints are imposed on the thermal history following nucleation. For instance, experimental studies have suggested that slow cooling or reheating after nucleation is necessary for the formation of BO chondrules (*21*, *34*). In BO textures formed at cooling rates exceeding 50 K hour^−1^, olivine develops a dendritic shape, whereas, in natural BO textures, olivine exhibits a smooth shape. The smooth shape is reproduced by ripening at relatively high temperatures (*34*). In our theoretical scenario, rapid cooling before nucleation is required for the formation of the global-scale rim; however, no specific restrictions are imposed on the cooling rate after nucleation. BO chondrules may have undergone textural ripening via reheating or slow cooling after the formation of the rim and bars under rapid cooling conditions.

The BO chondrules examined in this study account for only 3 to 4% of the total, and most of chondrules exhibit porphyritic textures (*9*). Porphyritic textures are believed to result from the overgrowth of residual crystals after incomplete melting and are fundamentally distinct from the crystal growth process described in this study. Therefore, the theoretical constraints proposed in this study cannot be generalized to all chondrules. According to previous experimental studies, the cooling rate experienced by porphyritic chondrules is thought to be slower than that of BO chondrules, ranging from 10 to 1000 K hour^−1^ (*11*). However, some reports suggest that porphyritic chondrules may have experienced rapid cooling at rates as high as 10^5^ K hour^−1^ (*35*–*37*). Our study highlights the importance of reexamining the potential for chondrule formation under such rapid cooling conditions.

Potential heating mechanisms capable of achieving such rapid cooling rates include planetary embryo bow shocks (*38*, *39*) and lightning (*40*) in optically thin environments. Alternatively, bow shocks generated by planetesimals or small protoplanets less than 100 km in size (*41*) could also fulfill the condition of extremely rapid cooling. These heating mechanisms were previously dismissed for not meeting observational constraints, especially, the cooling rate. In the future, theoretical verification of the formation conditions of chondrule-solidification textures may necessitate a reevaluation of the debated planetary formation environment within the early solar nebula.

## MATERIALS AND METHODS

### Compositional change inside the chondrule melt owing to evaporation

The time-dependent variation in melt composition owing to evaporation can be obtained by solving the diffusion equation, assuming negligible convective transport within the melt. In this study, we consider a two-dimensional circular melt, consistent with the two-dimensional nature of our numerical model (*18*). Although actual chondrules are three-dimensional spheres, this simplification has minimal impact on our discussion. Initially, the melt is assumed to have a uniform composition, *C*_0_. The melt radius is denoted as *a*, and the radial distance from the center is *r*. The composition distribution after a certain amount of time *t* has passed since the onset of evaporation is denoted by *C*(*r*, *t*). Assuming that mass loss owing to evaporation is proportional to the surface composition, the boundary condition at the melt surface is expressed as

$$-\;D\frac{\partial C}{\partial r}=\epsilon C.\;\left(r=a\right)$$ (1)

Here, *D* represents the diffusion coefficient, and ϵ denotes the evaporation rate. The time-dependent variation in composition distribution can be analytically solved for the two-dimensional axisymmetric diffusion equation, assuming constant values for D, ϵ, and *a* (*42*)

$$\frac{C\left(r,t\right)}{C_{0}}=2L\sum_{n=1}^{\infty }\frac{J_{0}\left(\beta_{n}r/a\right)}{\left(\beta_{n}^{2}+L^{2}\right)J_{0}\left(\beta_{n}\right)}e^{-\beta_{n}^{2}\mathrm{Dt}/a^{2}}$$ (2)

Here, L≡aϵ/D is referred to as the Peclet number and represents the normalized evaporation rate. The $\beta_{n}$ values are determined as the roots of the equation $J_{0}\left(\beta \right)-{\mathrm{LJ}}_{1}\left(\beta \right)=0$, where $J_{0}$ and $J_{1}$ are first-kind Bessel functions. By integrating Eq. 2, we obtain the time variation of the bulk composition, *M*_t_(*t*), which represents the total amount of the component within the melt

$$\frac{M_{t}\left(t\right)}{M_{t}\left(0\right)}=4L^{2}\sum_{n=1}^{\infty }\frac{e^{-\beta_{n}^{2}\mathrm{Dt}/a^{2}}}{\beta_{n}^{2}\left(\beta_{n}^{2}+L^{2}\right)}$$ (3)

Figure S1 illustrates *C*(*r*, *t*) for various cases. For *L* = 10, a pronounced compositional gradient forms near the melt surface. This occurs as mass loss owing to evaporation substantially outweighs the diffusive transport within the melt. As time progresses, the diffusive transport depletes the component from the melt center, leading to near-complete depletion throughout the melt at $\mathrm{Dt}/a^{2}∼1$. Conversely, for *L* = 0.1, no local depletion region forms near the melt surface. This is because diffusion-driven homogenization within the melt dominates over evaporation. These results indicate that an evaporation rate corresponding to L≳1 is necessary to establish a strong local composition gradient near the melt surface.

In this study, we consider a melt with a uniform equilibrium composition at 2000 K before evaporation. The melt was simulated to undergo evaporation during cooling at a constant rate of *R*_c_ = 10 K s^−1^ to 1990 K. The Peclet numbers for FeO and SiO_2_ were set to *L* = 1.0 and 0.3, respectively. The cooling time was *t* = 1 s. The diffusion coefficients of FeO and SiO_2_ were assumed to be 1.0 × 10^−8^ and 1.0 × 10^−9^ m^2^ s^−1^, respectively (*18*), with the temperature dependence neglected owing to the small temperature range. The melt radius was set to *a* = 110 μm. Figure S2 shows the compositional distribution of each component at 1990 K. A substantial depletion of FeO is observed throughout the melt. In contrast, although the bulk SiO_2_ composition remains relatively unchanged, a notable depletion occurs near the melt surface, approaching the stoichiometric composition of olivine.

### Anisotropy reduction in the evaporation layer

In this study, anisotropy was incorporated into the solid-liquid interface free energy, σ, and model parameter, M, which governs the growth kinetics (*18*). The anisotropy was expressed as $\sigma \left(\theta \right)=\sigma_{0}a_{s}\left(\theta \right)$ and $M\left(\theta \right)=M_{0}a_{m}\left(\theta \right)$, where θ represents the angle between the normal direction of the solid-liquid interface and the crystal’s *c* axis. Here, $\sigma_{0}$ and $M_{0}$ are constants. The functions $a_{s,m}\left(\theta \right)$ describe the anisotropy, with a minimum value of $\frac{1}{1+\varepsilon_{s,m}}$ at a specific direction, where parameters $\varepsilon_{s,m}$ control the strength of the anisotropy. When $\varepsilon_{s,m}=0$, σ and M are isotropic. To reproduce the experimental olivine morphology, we adopted the parameter values $\varepsilon_{s}=0.5$ and $\varepsilon_{m}=20$, as in previous studies (*18*).

In this study, we considered the reduced anisotropy within the evaporation layer by assuming that $\varepsilon_{s,m}$ varies with distance *h* from the melt surface. In particular, we replaced $\varepsilon_{s,m}$ with the following expression, denoted as $\varepsilon \prime_{s,m}$

$$\varepsilon \prime_{s,m}=\varepsilon_{s,m}\left(1-e^{-h/\delta_{\text{evap}}}\right)$$ (4)

Here, $\delta_{\text{evap}}$ represents the depth from the melt surface where anisotropy reduction occurs. At the melt surface (h=0), the crystal is assumed to be completely isotropic ($\varepsilon \prime_{s,m}=0$), whereas anisotropic growth occurs at depths greater than approximately $\delta_{\text{evap}}$. In our numerical calculations, we used a value of $\delta_{\text{evap}}=5$ μm; however, the results remain largely unaffected by variations in this parameter.

Figure S3 displays the simulation results without considering anisotropy reduction. The seed crystals introduced on the right side of the melt exhibited preferential growth along the *c* axis. Owing to strong anisotropic growth kinetics, growth along the *b* axis was suppressed, resulting in the absence of rim formation along the melt surface.
