# Supplementary material for: Decoding the formation of barred olivine chondrules: Realization of numerical replication
Source: Sci Adv. 2025 May 23;11(21):eadw1187. doi: 10.1126/sciadv.adw1187 (PMC12101491; doi:10.1126/sciadv.adw1187)
Supplement: Supplementary file 1 — Figs. S1 to S3 Legend for movie S1 [file sciadv.adw1187_sm.pdf]

Science Advances
AAAS

Supplementary Materials for

Decoding the formation of barred olivine chondrules: Realization of numerical replication

Hitoshi Miura *et al.*

Corresponding author: Hitoshi Miura, miurah@nsc.nagoya-cu.ac.jp;
Tomoki Nakamura, tomoki.nakamura.a8@tohoku.ac.jp

Sci. Adv. **11**, eadw1187 (2025)
DOI: 10.1126/sciadv.adw1187

The PDF file includes:

Figs. S1 to S3
Legend for movie S1

Other Supplementary Material for this manuscript includes the following:

Movie S1

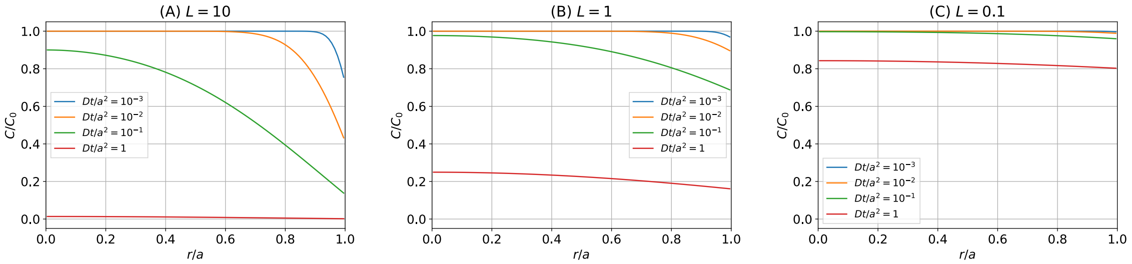

Figure S1: **Temporal evolution of composition distributions within a chondrule melt undergoing evaporation, under various evaporation conditions.** The horizontal axis represents the radial distance (r) from the melt center normalized by the melt radius (a), whereas the vertical axis shows the composition (C) normalized by the initial composition (C_0). The Peclet number, $L = a\epsilon/D$, characterizes the relative importance of the evaporation rate (ϵ) and diffusion coefficient (D). Different panels correspond to distinct Peclet number values: (**A**) $L = 10$, (**B**) $L = 1$, and (**C**) $L = 0.1$. Within each panel, the compositional profiles are shown at various dimensionless times, $Dt/a^2 = 10^{-3}$, 10^{-2}, 10^{-1}, and 1.

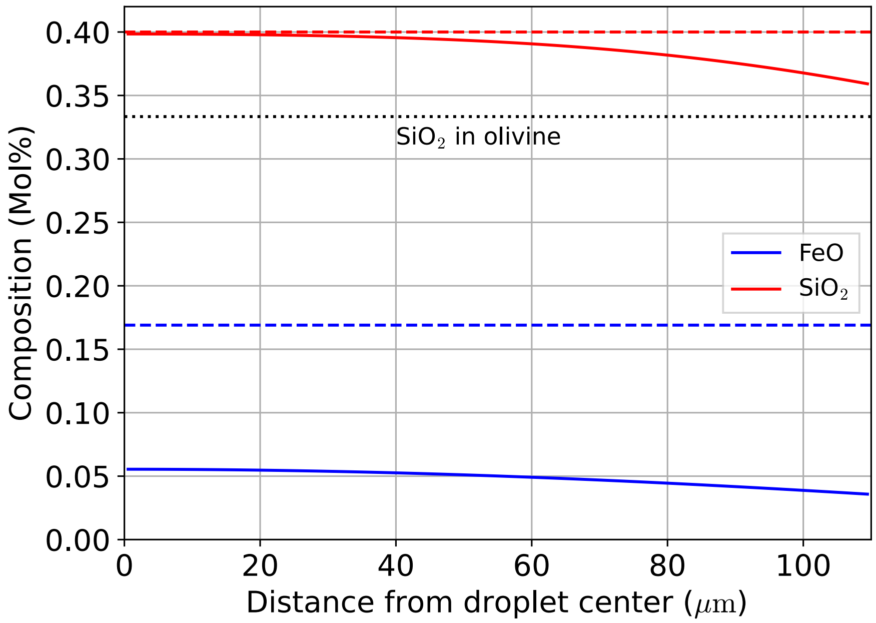

Figure S2: **Composition distributions of FeO and SiO_2 within the chondrule melt at the moment of seed-crystal introduction (solid line).** The dashed line represents the initial uniform composition of the melt before evaporation, with mole fractions of 0.169 and 0.400 for FeO and SiO_2, respectively. The dotted line indicates the stoichiometric SiO_2 composition of olivine.

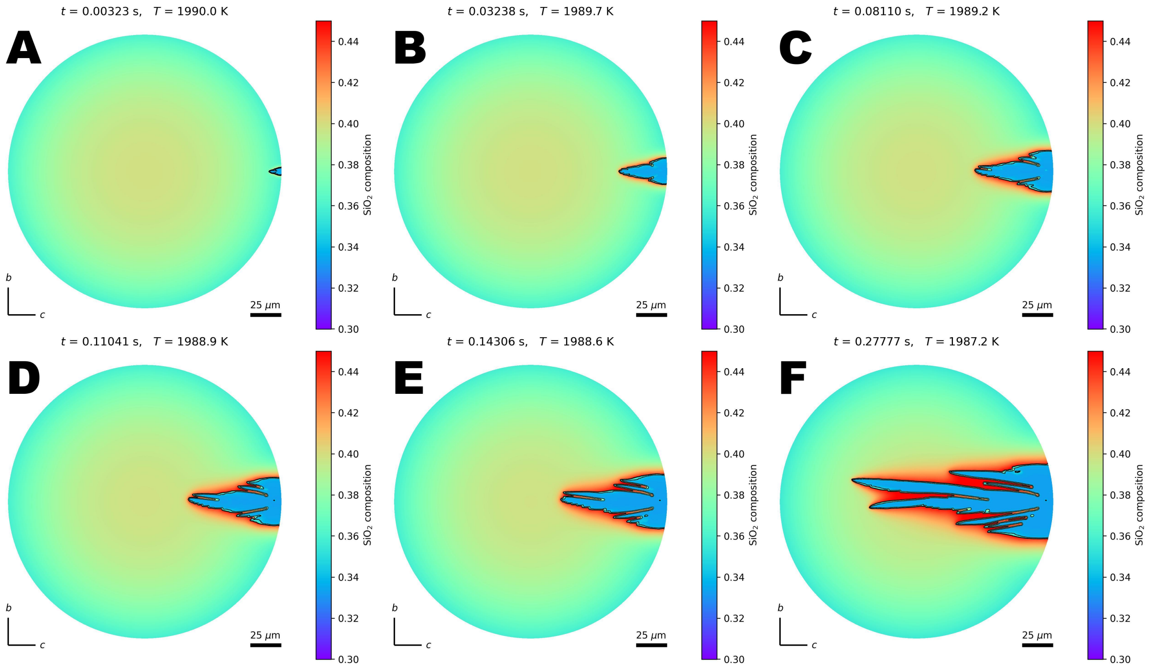

Figure S3: **Numerical simulation of olivine crystal growth in a chondrule melt.** The same as Figs. 2(A)–(F), but without considering anisotropy reduction in the evaporation layer. (**A**) Crystal growth immediately after the introduction of a seed crystal on the right side of the melt. (**B, C**) Crystal elongates along the *c*-axis and do not grow along the melt surface. (**D**) Crystal branches out into several protrusions. (**E, F**) Each protrusion elongates along *c*-axis.

Caption for Movie S1. Numerical simulation of olivine crystal growth in a chondrule melt.

Animated versions of Figs. 2(A)–(F).
